# Hyperglycemia-induced DNA damage response activates DNA-PK complex to promote endothelial ferroptosis in type 2 diabetic cardiomyopathy

**DOI:** 10.7150/thno.109514

**Published:** 2025-03-19

**Authors:** Cheng Luo, Chen Fang, Rongjun Zou, Jingwei Jiang, Miao Zhang, Teng Ge, Hao Zhou, Xiaoping Fan, Baoshi Zheng, Zhiyu Zeng

**Affiliations:** 1 Department of Cardiovascular Surgery, the First Affiliated Hospital of Guangxi Medical University, Nanning, 530021, China; 2 State Key Laboratory of Traditional Chinese Medicine Syndrome/Department of Cardiovascular Surgery, Guangdong Provincial Hospital of Chinese Medicine, the Second Affiliated Hospital of Guangzhou University of Chinese Medicine, the Second Clinical College of Guangzhou University of Chinese Medicine, Guangzhou 510120, Guangdong, China.; 3 State Key Laboratory of Dampness Syndrome of Chinese Medicine, Guangzhou 510120, Guangdong, China.; 4 The First Clinical Medical College of Guangxi Medical University, Nanning, 530021, China; 5 College of Pharmacy, Guangzhou University of Chinese Medicine, Guangzhou, 510405, China.; 6 University of Rochester Medical Center Rochester, NY, 601 Elmwood Ave, Rochester, NY 14642, United States.; 7 Department of Cardiology, The First Affiliated Hospital of Guangxi Medical University, 530021 Nanning, Guangxi, China.; 8 Guangxi Key Laboratory of Precision Medicine in Cardio-cerebrovascular Diseases Control and Prevention, 530021 Nanning, Guangxi, China.; 9 Guangxi Clinical Research Center for Cardio-cerebrovascular Diseases, 530021 Nanning, Guangxi, China.

**Keywords:** DNA damage response, DNA-PK complex, endothelial cells, type 2 diabetic cardiomyopathy

## Abstract

**Rationale:** Hyperglycemia-induced endothelial dysfunction is a hallmark of diabetic cardiomyopathy, yet the underlying molecular mechanisms remain incompletely understood. This study aimed to investigate how the DNA damage response (DDR) pathway regulates endothelial cell ferroptosis under hyperglycemic conditions, potentially revealing new therapeutic targets for mitigating cardiac damage in type 2 diabetes mellitus (T2DM).

**Methods:** We performed an integrated analysis of publicly available RNA sequencing datasets (GSE280770, GSE89475, GSE161931, CRA007245) to evaluate the role of DDR in hyperglycemia-induced endothelial dysfunction *in vitro* and *in vivo*, including in a T2DM mouse model. Key DDR and ferroptosis markers were validated in cardiac microvascular endothelial cells (CMECs) isolated from mice with streptozotocin (STZ)/high-fat diet (HFD)-induced T2DM, with and without treatment with the DNA-PK inhibitors NU7441 or M9831.

**Results:** Hyperglycemia induced a robust DDR in endothelial cells, characterized by the upregulation of DNA-PK complex genes (PRKDC, XRCC5, XRCC6) and increased markers of DNA damage (γH2AX, 8-oxo-dG). This was accompanied by increased expression of pro-ferroptotic genes (Tfrc, Acsl4, Ptgs2), decreased expression of anti-ferroptotic genes (Gpx4, Slc7a11), and elevated lipid peroxidation (MDA, 4-HNE). Pharmacological inhibition of DNA-PK mitigated these effects, reducing oxidative stress, lipid peroxidation, and endothelial permeability, while improving cardiac contractile and relaxation parameters.

**Conclusions:** Our findings implicate the DNA-PK complex as a key regulator of hyperglycemia-induced endothelial ferroptosis in T2DM cardiomyopathy. Targeting DNA-PK complex may represent a novel therapeutic strategy for mitigating microvascular dysfunction and cardiac decline in T2DM.

## Introduction

The coronary microcirculation in type 2 diabetes mellitus (T2DM) undergoes marked structural and functional changes, including capillary rarefaction and heightened vascular permeability, ultimately compromising myocardial oxygen and nutrient delivery and exacerbating ischemic injury and fibrosis 1, 2. Impaired endothelial function often occurs alongside insulin resistance in the small blood vessels that supply the heart muscle, diminishing endothelial nitric oxide (NO) bioavailability and contributing to perfusion deficits 3-5. Although strict glycemic control forms the cornerstone of therapy for microvascular complications in T2DM, these measures are not always sufficient 6. Clarifying the molecular mechanisms that drive hyperglycemia-induced damage to myocardial endothelial cells is thus crucial for identifying new therapeutic targets and guiding the development of endothelium-specific treatments 7.

A critical factor in maintaining genomic stability is the DNA damage response (DDR), which regulates cellular cycle, DNA regeneration, or apoptosis upon DNA insult 8, 9. Central to DDR are the ATM/ATR kinases, which phosphorylate downstream effectors, including p53, thereby halting cell cycle progression and potentially triggering apoptotic pathways 10, 11. In the context of diabetes, sustained hyperglycemia disrupts these protective processes, potentially contributing to the progression of diabetic complications through genomic instability. Elevated glucose levels induce DNA damage across various cell types, impacting both cancerous and healthy mammary epithelial cells 12, as demonstrated by heightened comet assay signals. This damage is further compounded by increased reactive oxygen species (ROS) 13, which can oxidatively modify DNA 14. Notably, hyperglycemia leads to the upregulation of multiple DDR genes, presumably as a compensatory mechanism, though the efficiency of DNA repair differs depending on cell type and physiological context 15. The genomic instability stemming from hyperglycemia is characterized by increased DNA damage and irregular expression of genes involved in inflammation and DNA repair, emphasizing the importance of glycemic management in curbing diabetic complications.

Ferroptosis, a controlled cell-death mechanism that depends on iron-mediated lipid peroxidation, is gaining prominence as a key contributor to diabetes and its sequelae. In diabetic retinopathy (DR), hyperglycemia promotes ferroptosis in retinal cells, intensifying vascular permeability and disrupting the blood-retinal barrier (BRB) 15. The aging retina, with increased iron deposition and compromised antioxidant defenses, is particularly vulnerable under hyperglycemic stress 15. Diabetic nephropathy shares similar characteristics, including prolonged inflammation and damage to the kidneys, in which iron overload and oxidative stress exacerbate ferroptotic cell death 16. Within the kidney, the SNHG1/miR-16-5p/ACSL4 axis has been highlighted as a regulator of hyperglycemia-induced ferroptosis 17. In diabetic cardiomyopathy, elevated intramyocardial lipid levels and iron accumulation further implicate ferroptosis as a driver of myocardial cell loss 18, underscoring its potential as a therapeutic target beyond traditional glucose-lowering approaches 18. Despite these advances, much remains unknown about the upstream modulators of ferroptosis and its role in myocardial endothelial cell dysfunction. Accordingly, our study aims to delineate how DDR regulates endothelial ferroptosis under hyperglycemic conditions, thereby illuminating potential targets for mitigating endothelial dysfunction and cardiac damage in T2DM.

## Methods

### Animal

Male C57BL/6J mice, aged eight to nine weeks (sourced from CLEA Japan Inc., Tokyo, or Japan SLC Inc., Shizuoka), were maintained in an environment with regulated temperature humidity, and photoperiod. They enjoyed unrestricted access to chow (MF, 12.8 kcal% fat; Oriental Yeast Co., Ltd.) and drinking water 19. Experimental procedures received ethical clearance from the Animal Experimentation Ethics Committees at the First Affiliated Hospital of Guangxi Medical University.

### Type 2 diabetes modeling

To model cardiomyopathy linked to type 2 diabetes, a combination of a high-fat diet (HFD) and low-dose streptozotocin (STZ) injections was used, adapting a previously published protocol 20. In summary, C57BL/6J mice consumed a specialized HFD (Research Diets, D12492) over 8 weeks to promote insulin resistance. Subsequently, mice were given STZ intraperitoneally (35 mg/kg; Sigma-Aldrich, S0130), daily for three days 21, 22. The animals remained on the HFD for a further 16 weeks. In contrast, the control group mice were fed a standard normal control diet (NCD) and given citrate buffer vehicle only 23. Mice were weighed and their blood glucose levels were monitored every two weeks. A diabetes diagnosis was confirmed when random blood glucose levels exceeded 16.7 mmol/L in two consecutive measurements. Echocardiography was used to verify the presence of diabetic cardiomyopathy. At the study's conclusion, whole blood samples were obtained by cardiac puncture, using heparin (10 U/mL) to prevent coagulation, and plasma was separated. Hearts underwent PBS perfusion to eliminate blood, were then weighed, and either prepared for histological assessment or stored at -80 °C for future molecular and biochemical assays 24.

### Cardiac ultrasound imaging

Cardiac ultrasound imaging on mature mice was executed following a modified version of established techniques. Animals were rendered unconscious using 2% isoflurane inhalation. Following anesthesia, abdominal fur was carefully cleared away with depilatory cream. A thin layer of warmed ultrasound transmission gel was spread across the exposed skin 25. The transducer was then brought into gentle contact with the gel, and slowly advanced towards the skin surface until the myocardium was identified. Image sequences were recorded when the cardiac structures were clearly visible on the monitor 26, 27.

### Single-nucleus RNA sequencing (snRNA-seq) data re-analysis

To investigate the translational relevance of the transcriptional alterations found in our mouse model, we obtained and re-processed a publicly available single-nucleus RNA-seq dataset (CRA007245). This dataset comprised 32,585 cardiac cells: 16,490 from six control individuals and 16,095 from six HFD/STZ-treated diabetic mice. In short, quality control (QC) steps removed genes detected in fewer than three cells. Cells were retained if they had between 500 and 4000 unique genes detected, and a mitochondrial gene percentage below 20% of total UMI counts. After QC, cells meeting these criteria were included in subsequent bioinformatic analyses 28, 29. Furthermore, data normalization was carried out with the NormalizedData function, and highly variable genes were detected via the FindVariableFeatures function. Data from multiple samples was integrated using the Harmony package's integration function. To identify marker genes for each cluster, Wilcoxon rank-sum tests with Benjamini-Hochberg false discovery rate (FDR) correction were applied, considering an adjusted p-value threshold of less than 0.01, implemented through the FindAllMarkers function. Cell type annotation was performed through systematic cross-referencing with CellMarker 2.0 (v2.0, http://117.50.127.228/CellMarker/), a manually curated repository containing 83,361 tissue-cell type-marker entries validated by 48 sequencing platforms including 10X Chromium and Smart-seq2 30, 31.

### 8-oxo-dG measurement

DNA (100 ng) was denatured and coated onto a 96-well plate. 8-oxo-dG levels were detected using a monoclonal antibody specific to 8-oxo-dG 20, 32.

### FITC-dextran permeability assay

Endothelial permeability was assessed using FITC-dextran (70 kDa, Sigma). CMECs were seeded on Transwell inserts (3 μm pores, Corning) and grown to confluence. The upper chamber was supplemented with FITC-dextran at a concentration of 1 mg/mL 33. Following a 1-hour incubation, the fluorescence intensity in the lower chamber was quantified using a microplate reader at excitation and emission wavelengths of 485 nm and 535 nm, respectively. The data were then normalized relative to the control group 34, 35.

### Transendothelial electrical resistance (TER)

TER measurements were obtained using the Electric Cell-Substrate Impedance Sensing (ECIS) System from Applied Biophysics. CMECs were seeded on 8W10E+ electrode arrays. Impedance was monitored at 4,000 Hz every 10 min until stabilization. TER values (Ω·cm²) were calculated using ECIS software 36, 37.

### Transcriptome sequencing (RNA-seq) data processing

To investigate changes in gene expression within endothelial cells exposed to hyperglycemia, we accessed and re-evaluated three RNA-seq datasets (GSE280770, GSE89475, and GSE161931). Gene transcript abundance was quantified as Read Counts. Differential gene expression analysis was conducted using these abundance estimates 38, 39. Differential expression analysis was performed using the exactTest function from the edgeR package to determine the statistical significance of the observed changes. The edgeR package's exactTest function was employed to assess the statistical significance of differential expression, along with fold-change calculations. The null hypothesis tested was that gene expression was equivalent between groups 40, 41.

### Isolation and culture of cardiac microvascular endothelial cells (CMECs)

CMECs were obtained from two-week-old mice, adapting a previously established protocol. The isolated CMECs were maintained in MEM medium supplemented with 10% heat-inactivated fetal bovine serum (FBS), 1% Penicillin/Streptomycin (P/S), and 1% L-Glutamine (Life Technologies). To mimic hyperglycemic conditions, CMECs were exposed to high glucose (HG, 30 mmol/L) in the culture medium for 48 hours. Control CMECs were cultured in medium containing normal glucose levels (NG, 5.5 mmol/L) 42, 43.

### Cellular viability and caspase activity assays

To evaluate the cytotoxic effects of high glucose on CMECs, CellToxGreen (G8741; Promega) and RealTime-Glo™ MT Cell Viability (G9711; Promega) assays were employed, following the manufacturer's protocols. After experimental treatments, culture medium was supplemented with the viability and toxicity indicator dyes. Luminescence and fluorescence signals were quantified using a Varioskan microplate reader 44. Caspase-3 activity was assessed using the Caspase-3 kit from Promega, according to the manufacturer's instructions 45, 46.

### ELISA

Malondialdehyde (MDA) levels were measured using the TBARS Assay Kit from Cayman Chemical. CMEC lysates were mixed with thiobarbituric acid (TBA) and the absorbance was quantified at a wavelength of 532 nm. 4-Hydroxynonenal (4-HNE) was detected using an ELISA Kit 47. GPX4 levels were determined using the Glutathione Peroxidase 4 (GPX4) ELISA Kit. Chk2 and γH2AX activities was quantified using the ELISA Kit 48, 49.

### Intracellular reactive oxygen species (ROS) measurement

To quantify intracellular ROS levels, cells were plated in culture dishes and then incubated with a cell-permeable probe 2',7'-dichlorofluorescin diacetate (DCFDA, final concentration 10 µM) for 45 minutes at 37°C under light-protected conditions 50. After incubation, cells were rinsed twice with phenol red-free medium containing 10% FBS. The resulting fluorescence from DCFDA oxidation was then quantified using a Varioskan microplate reader (5187139; ThermoFisher Scientific) at 37°C 51, 52.

### Subpopulation-specific differential expression analysis in snRNA-seq

The analysis aimed to pinpoint genes with differential expression between subpopulations exhibiting high and low DDR of capillary endothelial cells in diabetic cardiomyopathy model, we used the FindMarkers function in Seurat (version 4.0.1) 53. Statistical significance was established using a Benjamini-Hochberg adjusted p-value cutoff of <0.05, combined with an absolute log2 fold change of >0.3 54. Gene Set Enrichment Analysis (GSEA) was conducted via log2FC-ranked gene lists to further elucidate the molecular mechanisms underlying DDR heterogeneity in diabetic cardiomyopathy.

### Quantitative real-time PCR (qPCR)

Nucleic acids were extracted from experimental specimens employing TRIzol solution (Thermo Fisher Scientific). RNA concentration and integrity were verified through spectrophotometric measurement (NanoDrop 2000, Thermo Fisher Scientific). Complementary DNA synthesis was initiated with two micrograms of purified RNA template 55. Target amplification cycles were executed on a LightCycler 480 II platform (Roche) employing sequence-specific oligonucleotides. Experimental workflows strictly complied with reagent provider specifications 56. Primer nucleotide sequences are cataloged in Supplementary Table 1.

### Statistical methods

Data are expressed as the mean value accompanied by the standard deviation (SD). Statistical comparisons between two groups were conducted using Student's t-test for data with a normal distribution, while the Mann-Whitney U test was employed for data that did not meet normality assumptions. When comparing three or more groups, one-way analysis of variance (ANOVA) was used. Statistical significance was defined as a p-value of less than 0.05.

## Results

### High glucose exposure triggers a robust DNA damage response in mouse endothelial cells

**Figure 1:**
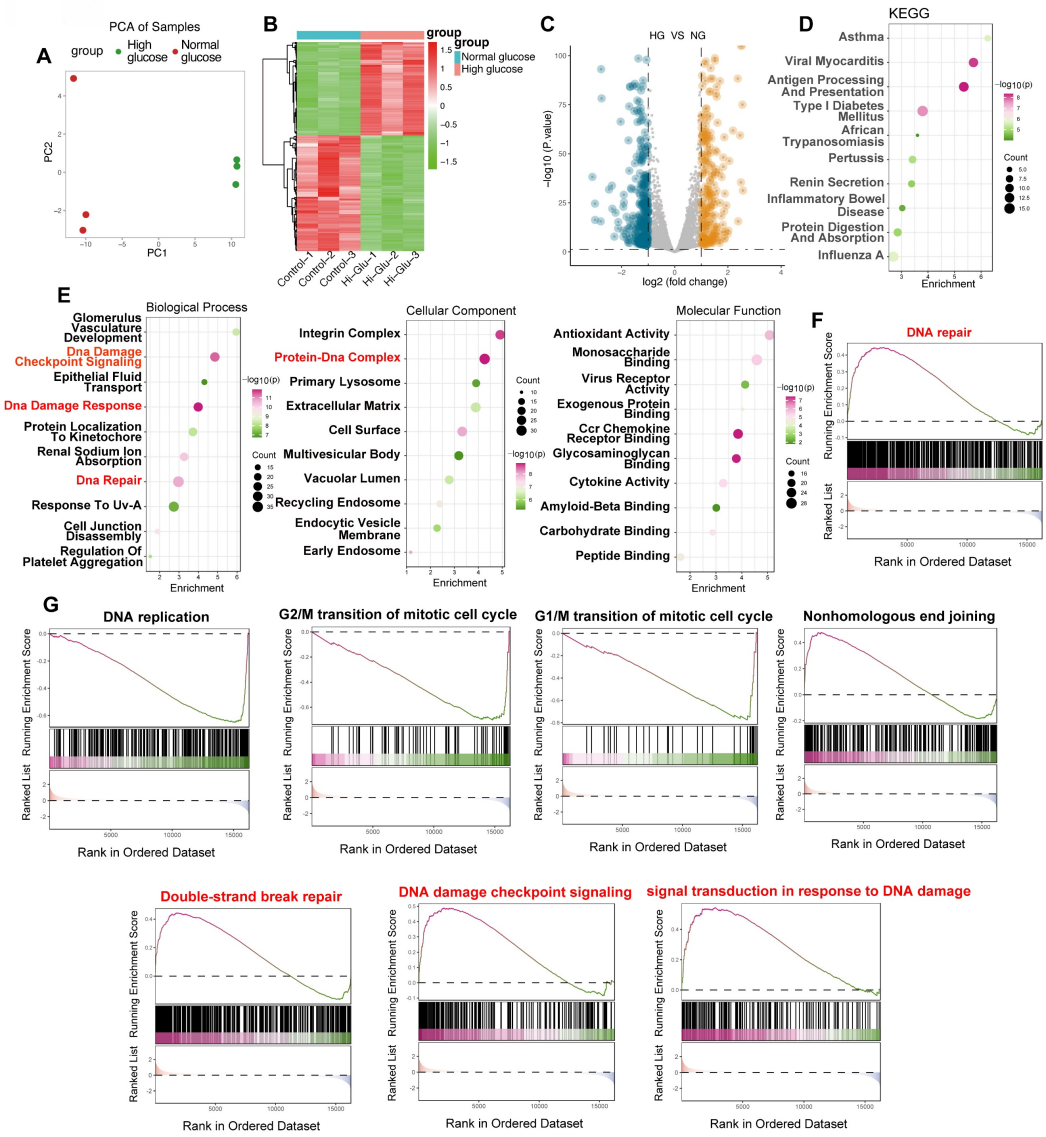
** High glucose elicits a robust DNA damage response in endothelial cells: bioinformatic analysis of the GSE280770 dataset.** (A) Principal Component Analysis (PCA). PCA plot illustrating the clear separation between samples treated with high glucose (red) and those exposed to normal glucose (green). PC1 and PC2 denote the first two principal components. (B) Heatmap of Differential Gene Expression. Heatmap visualizing genes that are differentially expressed between high glucose and normal glucose conditions. Upregulation is indicated in red and downregulation in green. Samples are labeled as Control-1 to Control-3 and Hi-Glu-1 to Hi-Glu-3, respectively. (C) Volcano Plot. Volcano plot displaying the distribution of differentially expressed genes between high glucose (HG) and normal glucose (NG) groups. The x-axis represents the log₂ fold change, while the y-axis shows the -log₁₀(p-value). Blue dots denote downregulated genes and orange dots denote upregulated genes. (D) KEGG pathway enrichment analysis. Bubble plot depicting enriched KEGG pathways. The x-axis indicates the degree of enrichment, the y-axis lists the enriched terms, bubble size reflects the gene count, and the color gradient corresponds to -log₁₀(p-value). Pathways pertinent to DNA damage and repair (e.g., "Type I Diabetes Mellitus") are highlighted. (E) Gene Ontology (GO) enrichment analysis. Bubble plots presenting enriched GO terms across three categories: Biological Process, Cellular Component, and Molecular Function. The x-axis shows enrichment scores, while the y-axis lists the GO terms. Bubble sizes correspond to gene counts and colors to -log₁₀(p-value). Key terms such as “DNA Damage Checkpoint Signaling,” “DNA Damage Response,” and “DNA Repair” (Biological Process) and “Protein-DNA Complex” (Cellular Component) are emphasized. (F) Gene Set Enrichment Analysis (GSEA) of DNA Repair. GSEA plot demonstrating the enrichment of the “DNA repair” gene set in the high glucose group. The y-axis shows the running enrichment score, and the x-axis presents the ranked gene list; black bars indicate the positions of genes within the set. (G) GSEA of additional DNA damage-related pathways. GSEA plots for pathways including “DNA replication,” “G2/M transition,” “G1/M transition,” “Nonhomologous End Joining,” “Double-Strand Break Repair,” “DNA Damage Checkpoint Signaling,” and “Signal Transduction in Response to DNA Damage.” The plots share the same format as in (F).

To evaluate the influence of elevated glucose on endothelial cell DNA damage response (DDR), we conducted a comprehensive bioinformatic analysis of the GSE280770 dataset, which contains gene expression profiles from murine skin endothelial cells—an appropriate model for *in vitro* studies. Cells were cultured under either normoglycemic (5 mmol/L glucose) or hyperglycemic (20 mmol/L glucose) conditions for a period of 48 hours to assess the effects of high glucose levels. Principal component analysis (PCA) revealed a clear separation between the two groups (Figure 1A), indicating significant changes in global gene expression patterns triggered by high glucose. A heatmap further visualized differential gene expression (Figure 1B), and a volcano plot (Figure 1C) quantified substantial gene regulation, highlighting both upregulated and downregulated genes. To explore the underlying mechanisms, we performed KEGG and Gene Ontology (GO) enrichment analyses. Both analyses identified a notable enrichment of pathways and terms related to DNA damage and repair. Specifically, the GO Biological Process category was enriched with terms such as "DNA Damage Checkpoint Signaling," "DNA Damage Response," and "DNA Repair" (Figure 1E), while the Cellular Component category highlighted the "Protein-DNA Complex." GSEA further confirmed significant enrichment of the "DNA repair" pathway in high glucose conditions (Figure 1F), with additional pathways associated with DNA damage and repair, including "DNA replication," cell cycle checkpoints (G1/M and G2/M transitions), "Nonhomologous End Joining," "Double-Strand Break Repair," and "DNA Damage Checkpoint Signaling" (Figure 1G). These findings strongly suggest that high glucose exposure triggers a potent DDR in endothelial cells, marked by altered gene expression and activation of critical DNA repair pathways.

### Hyperglycemia activates dna damage response pathways in a type 2 diabetes mouse model

**Figure 2:**
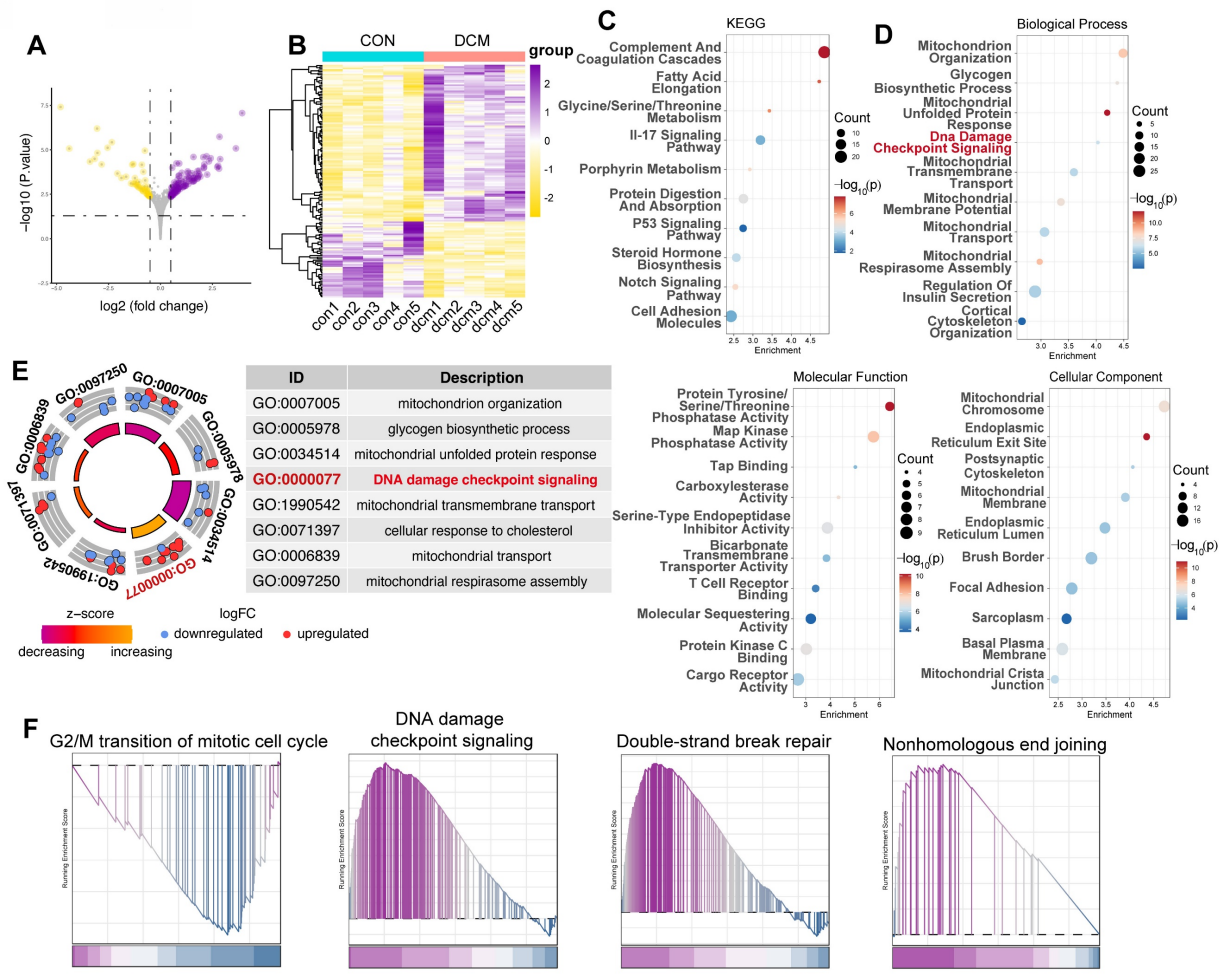
** Transcriptomic analysis reveals activation of DNA damage response pathways in a type 2 diabetes mouse model (GSE161931).** (A) Volcano plot displays differentially expressed genes (DEGs) between control mice and db/db mice. The x-axis indicates the log₂ fold change, and the y-axis represents -log₁₀(p-value). (B) Heatmap illustrates normalized gene expression (z-scores) for DEGs between control mice and db/db mice, with yellow signifying lower expression and purple higher expression.(C) KEGG pathway enrichment. Bubble plot showing KEGG pathway enrichment among DEGs; dot size reflects gene number, and color indicates -log₁₀(p-value). Pathways such as the “p53 signaling pathway” are underscored. (D) GO enrichment analysis. Bubble plot of DEGs, segregated into Biological Process, Molecular Function, and Cellular Component categories. The x-axis shows enrichment, the y-axis lists GO terms, and the color gradient represents -log₁₀(p-value). (E) Circle plot summarizes selected enriched GO terms, notably “DNA damage checkpoint signaling” (GO:0000077). The inner circle indicates the z-score and the outer circle displays the log₂ fold change for each related gene. (F) GSEA plots depicts enrichment for gene sets associated with “G2/M transition,” “DNA damage checkpoint signaling,” “Double-Strand Break Repair,” and “Nonhomologous End Joining.” The plot shows the running enrichment score (y-axis) across the ranked gene list (x-axis), with black vertical lines marking gene positions.

To further validate our *in vitro* findings and evaluate the *in vivo* relevance of DDR in diabetic cardiomyopathy, we analyzed the GSE161931 dataset, which contains RNA sequencing data from heart tissue of *Mus musculus*, comparing db/db mice with healthy mice. Differential gene expression analysis revealed significant transcriptional changes in the hearts of db/db mice compared to control BKS mice. 103 genes were upregulated, while 50 genes were downregulated, indicating a substantial impact of diabetic cardiomyopathy on cardiac gene expression (Figure 2A,B). Consistent with our hypothesis, pathway enrichment analysis using KEGG and GO databases showed a significant overrepresentation of key pathways associated with DNA damage and repair, including the p53 signaling pathway (Figure 2C) and DNA damage checkpoint signaling (Figure 2D, E). Moreover, GSEA demonstrated significant enrichment of pathways associated with G2/M cell cycle arrest, DNA damage checkpoint signaling, double-strand break repair, and nonhomologous end joining (Figure 2F), reinforcing the activation of DDR pathways in endothelial cells exposed to a diabetic environment.

### High glucose induces differential gene expression and activates DDR pathways in human iPSC-derived endothelial cells under hyperglycemic stress

**Figure 3:**
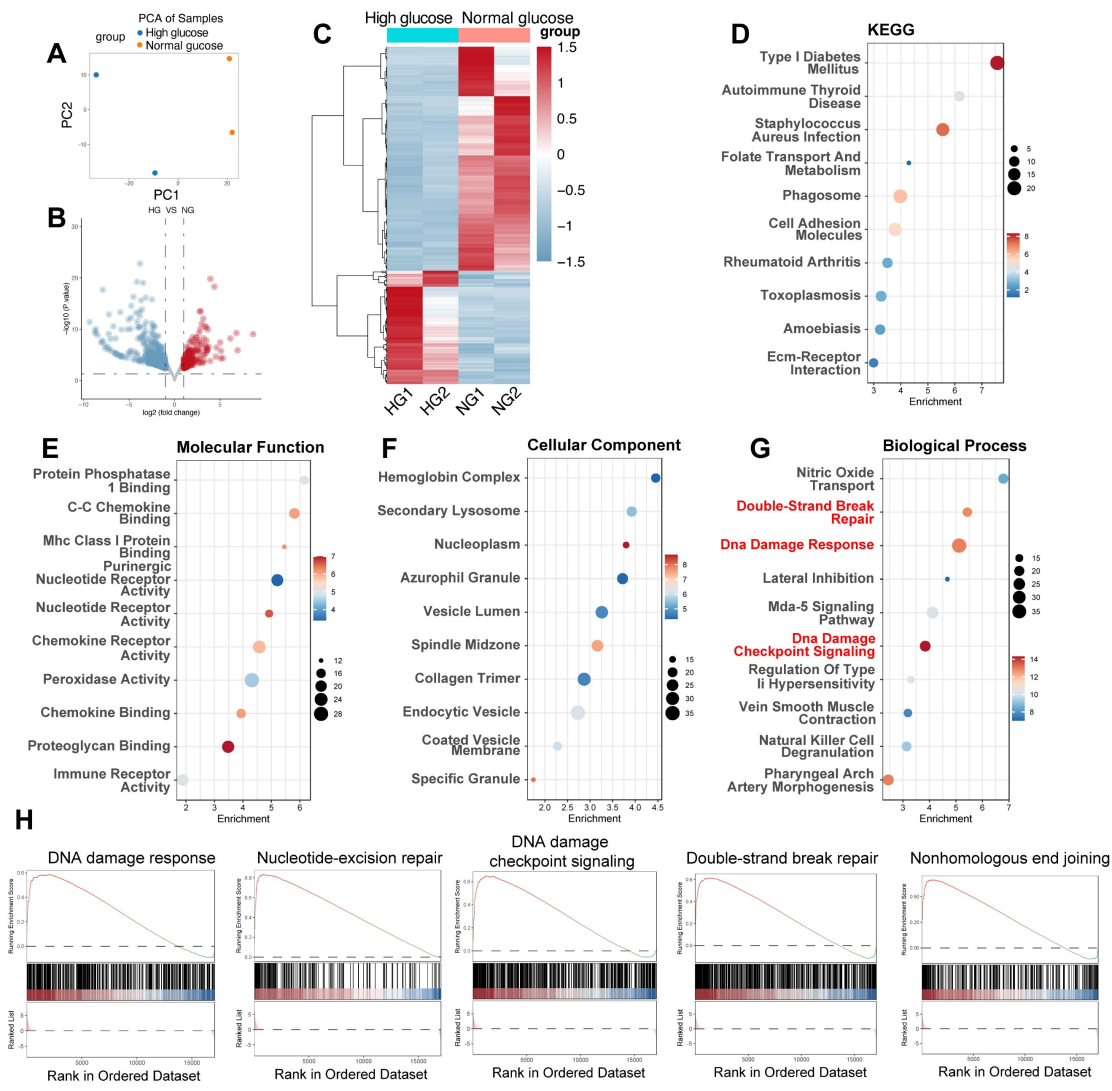
** High glucose triggers differential gene expression and activates DNA damage response pathways in human iPSC-derived endothelial cells.** (A) PCA plot. Each point represents a sample from high glucose (HG) and normal glucose (NG) groups, illustrating the separation based on gene expression profiles.(B) Volcano plot displays DEGs between HG and NG groups; upregulated genes appear in red and downregulated in blue, with the x-axis showing log₂ fold change and the y-axis -log₁₀(p-value). (C) Heatmap shows normalized expression levels for DEGs across HG and NG samples; red denotes higher expression and blue lower expression. (D) KEGG enrichment. Bubble plot indicating enriched KEGG pathways among DEGs, with “Type I Diabetes Mellitus” highlighted. (E-G) GO enrichment. Bubble plots for Molecular Function (E), Cellular Component (F), and Biological Process (G) with key terms related to DNA damage (e.g., “DNA damage response,” “DNA damage checkpoint signaling,” “Double-Strand Break Repair”) emphasized in red. (H) Display enrichment of gene sets corresponding to “DNA damage response,” “Nucleotide-Excision Repair,” “DNA Damage Checkpoint Signaling,” “Double-Strand Break Repair,” and “Nonhomologous End Joining.”

To corroborate our findings in mouse models and examine the transcriptional response of human endothelial cells to a hyperglycemic environment, we analyzed the GSE89475 dataset. This dataset includes RNA sequencing data from human iPSC-derived endothelial cells cultured under normal glucose (NG, 17mM) or diabetic (DI) conditions, simulating a diabetic milieu with high glucose (75mM), TNF-α (1ng/mL), and IL-6 (1ng/mL). PCA was employed to provide a visual representation of the overall transcriptional variations between NG and HG conditions (Figure 3A). Differential gene expression analysis revealed significant alterations in the transcriptome under DI conditions compared to NG (Figure 3B-C). 176 genes were upregulated, while 348 genes were downregulated, reflecting a comprehensive transcriptional response to the hyperglycemic environment (Figure 2B). This *in vitro* model, as previously reported, displays hallmark features of diabetic vasculopathy, including basement membrane thickening, providing a relevant context for studying the molecular mechanisms of diabetic complications. Consistent with our hypothesis, pathway enrichment analysis using KEGG and GO databases revealed significant enrichment of pathways related to DNA damage and repair, including the terms of “Double-Strand Break Repair”, “DNA damage Response” and “DNA damage checkpoint signaling” (Figure 3D-G). Additionally, GSEA confirmed the activation of DNA damage response pathways, including "DNA damage response," "Nucleotide-excision repair," "DNA damage checkpoint signaling," "Double-strand break repair," and "Nonhomologous end joining" (Figure 3H). These results provide strong evidence that DNA damage and repair mechanisms are activated in heart tissue under diabetic conditions.

### Single-cell RNA-seq reveals DNA damage and repair in db/db diabetic heart capillary endothelium

**Figure 4:**
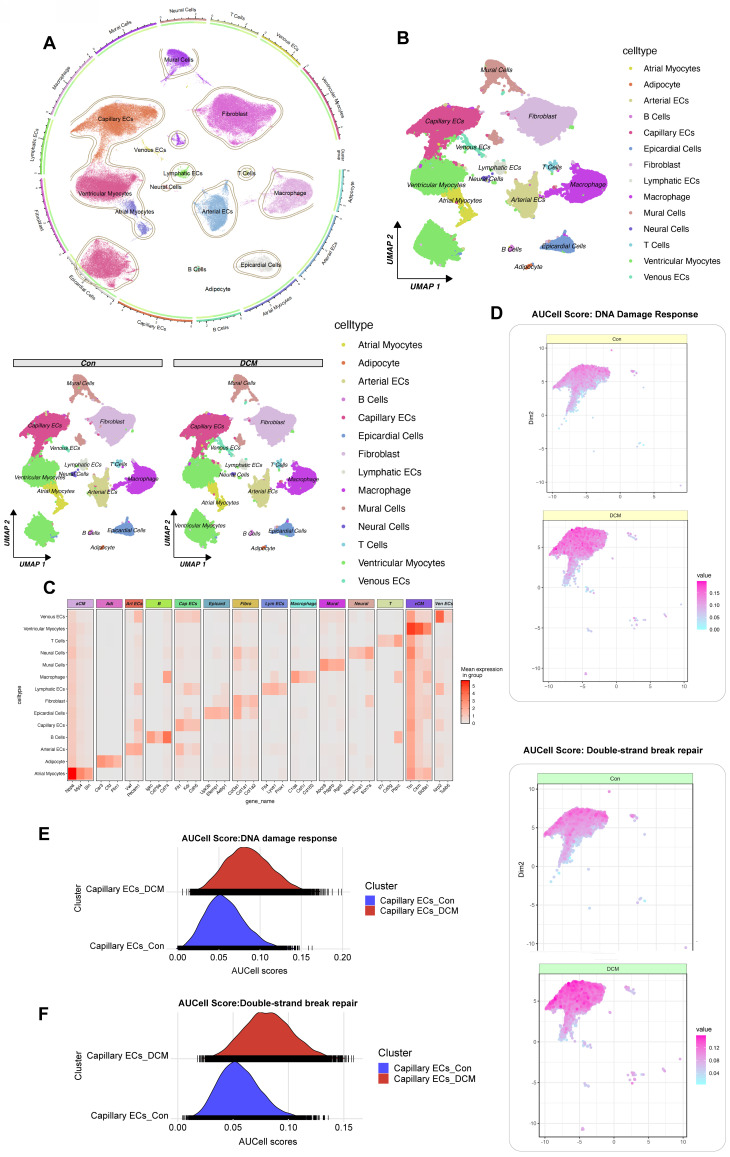
** Single-cell RNA sequencing analysis of heart tissue from a db/db mouse model reveals activation of DDR and DSB repair pathways in capillary endothelial cells.** (A) UMAP Visualization displays all identified cell clusters from snRNA-seq of nuclear fractions from cardiac cells; A circular plot on the left provides an alternative visualization of cluster relationships and the proportional distribution of each subtype between control and DCM groups. (B) UMAP by condition. UMAP plots for control (db/m) and diabetic (db/db) samples. (C) Heatmap of marker genes. The mean expression levels of selected marker genes for each cell type (e.g., aCM, Art ECs, Cap ECs, vCM). (D) Featureplot of AUcell scores for DDR. Featureplot of AUCell scores for the DDR pathway in control and db/db samples, with higher scores indicating greater activity. (G) Featureplot of AUcell scores for DSB Repair. Featureplot of AUCell scores for the DSB repair pathway in control and diabetic samples. (E) Ridge plot for DDR in capillary ECs. The distribution of DDR activity between control (blue) and diabetic (red) capillary ECs. (F) Ridge plot for DSB Repair. Double-strand break repair pathway activity in capillary ECs.

To dissect the cellular heterogeneity of DDR in diabetic cardiomyopathy, we performed single-cell RNA sequencing (snRNA-seq) on nuclear fractions from all cardiac cells, allowing us to assess the heterogeneity of cell populations and transcriptional changes in response to HFD/STZ-induced diabetes. The analysis included a total of 32,585 cardiac cells, comprising 16,490 cells from 6 healthy control mice and 16,095 cells from 6 mice with HFD and STZ-induced diabetes, 25 transcriptionally distinct pre-clusters were identified, which were further categorized into 14 distinct cell populations based on the expression of cell-specific markers and enriched genes. Cell subtype identification based on marker genes revealed a diverse range of cardiac cell types, including capillary endothelial cells (Capillary ECs), cardiomyocytes, fibroblasts, and immune cells. The proportional distribution of each cell subtype was quantified between control and model groups, and group-specific UMAP visualizations were generated to illustrate the compositional differences. (Figure 4A, B). Canonical marker gene expression analysis confirmed the identities of these clusters (Figure 4C). We then focused on Capillary ECs, a critical population involved in diabetic microvascular complications. Using AUCell, we assessed the activity of DDR and double-strand break repair pathways in the Capillary ECs subpopulation. A marked increase in AUCell scores for both DDR and DSB repair was observed in capillary ECs from db/db diabetic mice compared to db/m control mice (Figure 4D-G). Specifically, the distribution of AUCell scores for DDR (Figure 4E) and DSB repair (Figure 4F) shifted towards higher values in the diabetic capillary ECs, indicating enhanced pathway activity. These findings, derived from a single-cell transcriptomic analysis of a well-characterized db/db mouse model of diabetic cardiomyopathy, provide compelling evidence that DDR and repair pathways are selectively activated in cardiac capillary endothelial cells under diabetic conditions.

### DNA-PK complex expression correlates with enhanced DNA damage response in diabetic cardiac endothelial cells

**Figure 5:**
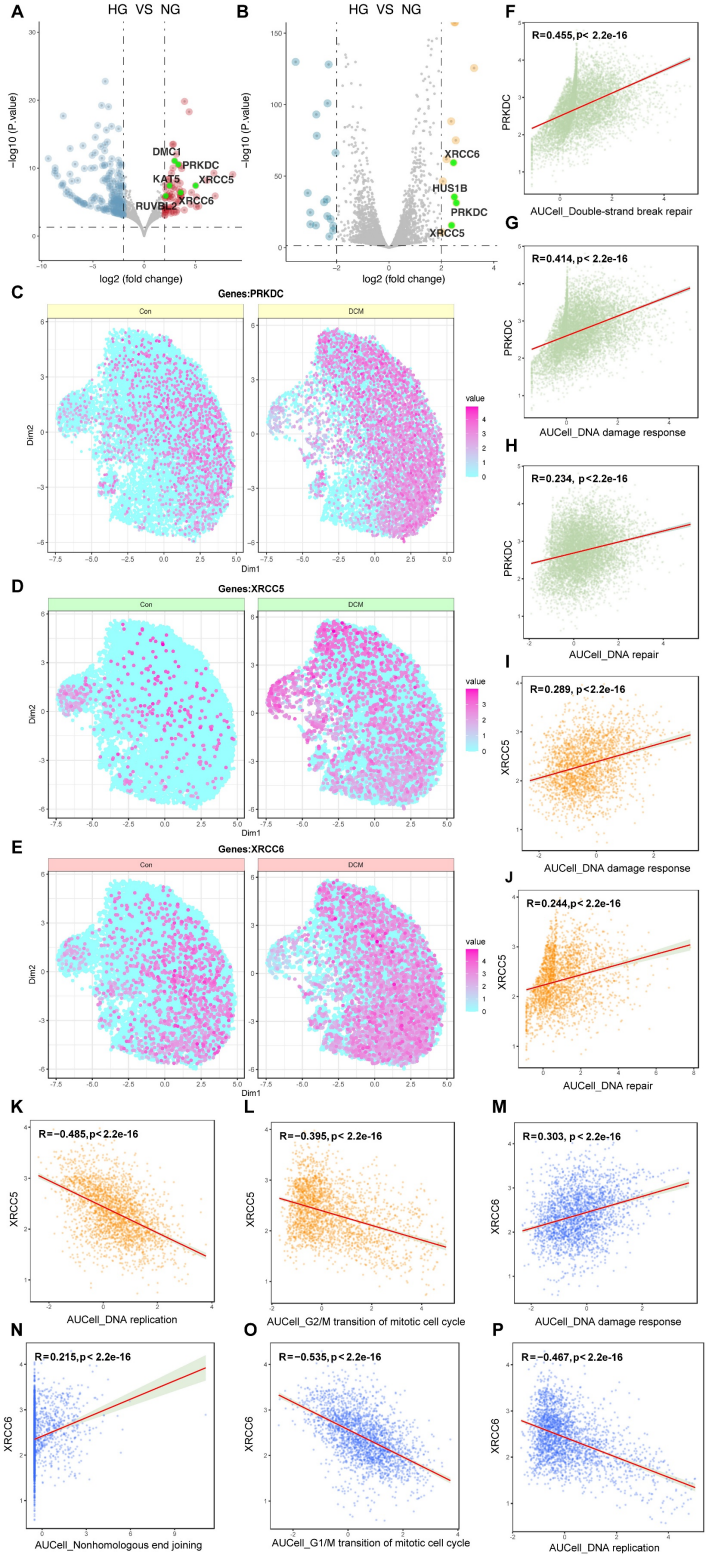
** Multi-faceted analysis of DDR and DNA repair genes in high glucose/diabetic cardiomyopathy conditions.** (A-B) Differential gene expression. Volcano plots from the GSE89475 and GSE280770 datasets; genes highlighted in green (e.g., PRKDC, XRCC5, XRCC6) are significantly upregulated and belong to the “DNA damage response” GO term. (C-E) Single-cell expression. Visualizes the expression of PRKDC, XRCC5, and XRCC6 in control and diabetic cardiomyopathy conditions (CRA007245 dataset) using color intensity as a proxy for expression levels. (F-P) Correlation analysis. Scatter plots depicting correlations between the expression levels of PRKDC, XRCC5, or XRCC6 and AUCell scores for various DNA repair and cell cycle pathways. Each point represents a single cell; the red regression line, Pearson correlation coefficient (R), and p-value are shown to test the association between DNA-PK complex gene expression and pathway activity.

To further investigate the molecular regulators of DDR in high glucose-induced endothelial dysfunction, we performed a cross-dataset analysis integrating bulk RNA-sequencing data from *in vitro* (GSE89475) and *in vivo* (GSE280770) models, alongside single-nucleus RNA-sequencing data from a diabetic cardiomyopathy mouse model (CRA007245). Initial screening of the GSE89475 dataset revealed significant upregulation of genes relative to the DNA damage response, including those encoding components of the DNA-dependent protein kinase (DNA-PK) complex—PRKDC, XRCC5, and XRCC6—across both datasets (Figure 5A, B). To assess cell-type specificity, we analyzed the CRA007245 dataset, which includes single-nucleus RNA-seq data from the hearts of diabetic and non-diabetic mice. UMAP visualization revealed increased expression of PRKDC, XRCC5, and XRCC6 across multiple cell types within the diabetic heart, with a particularly pronounced upregulation in capillary endothelial cells (Figure 5C-E). Correlation analysis within the CRA007245 dataset further showed a strong and statistically significant positive correlation between PRKDC expression and AUCell scores for both DSB repair and overall DDR activity (Figure 5F, G). A positive correlation, though weaker, was observed between XRCC5 expression and DNA damage response/repair pathways (Figure 5H-J). Conversely, XRCC6 expression exhibited negative correlations with DNA replication and G2/M transition, but a positive correlation with DDR (Figure 5K-P). These findings indicate that the DNA-PK complex, particularly PRKDC, plays a critical role in the amplified DDR observed in cardiac endothelial cells under diabetic conditions.

### Activation of DDR in CMECs under diabetic conditions

**Figure 6:**
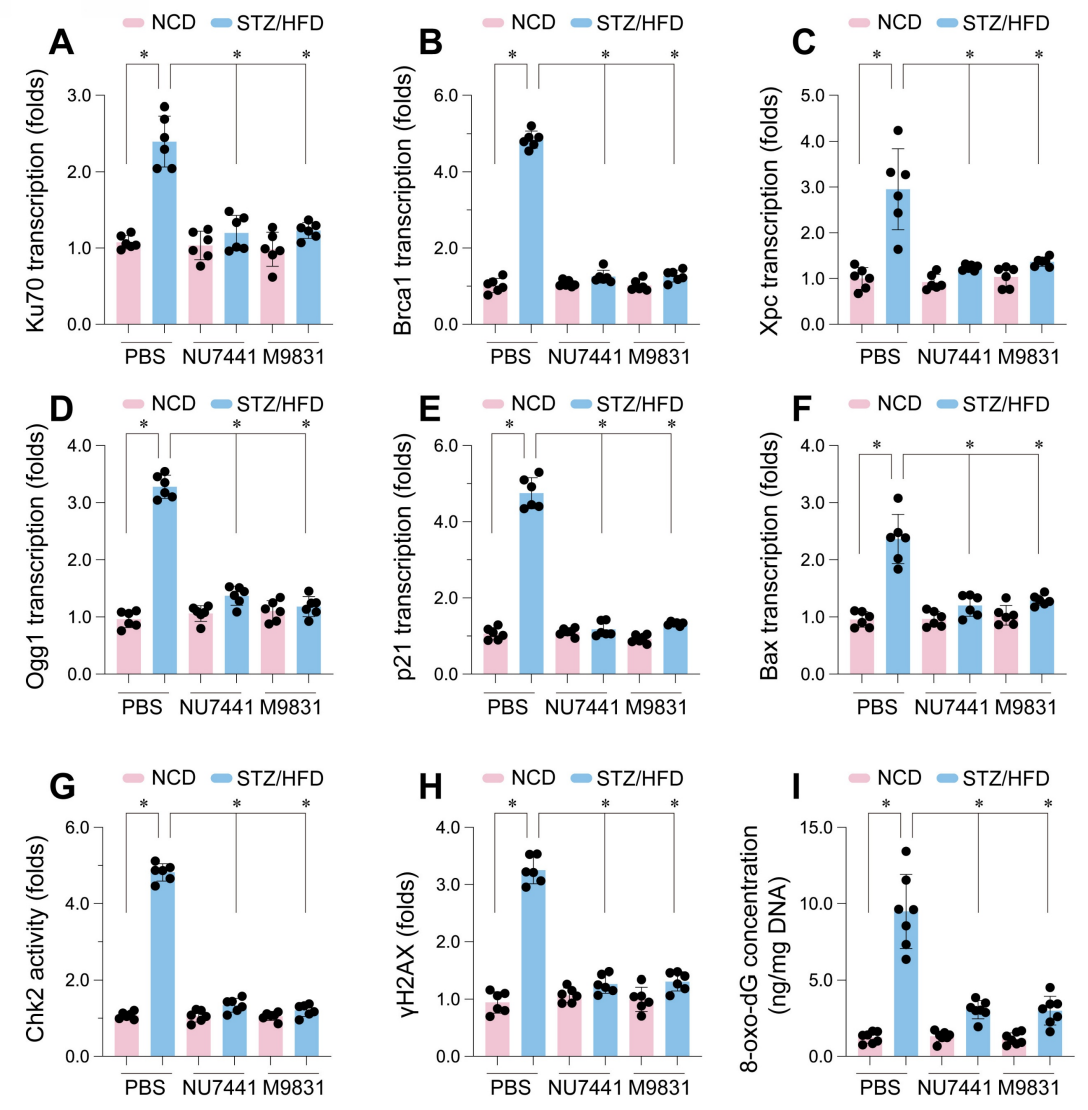
** DNA-PK complex inhibition attenuates DNA damage and DDR pathway activation in CMECs from STZ/HFD-induced diabetic mice.** Cardiac microvascular endothelial cells (CMECs) were isolated from mice fed a normal control diet (NCD) or subjected to streptozotocin (STZ) and a high-fat diet (HFD) to induce type 2 diabetes. Mice received either PBS (vehicle), NU7441, or M9831 (DNA-PK inhibitors). (A-F) mRNA expression levels of key genes were quantified and normalized. (A) Ku70, (B) Brca1, (C) Xpc, (D) Ogg1, (E) p21, and (F) Bax. (G) Chk2 Activity was determined by ELISA. (H) Measurement of phosphorylated H2AX as a marker for double-strand breaks. (I) Quantification of oxidative DNA damage. Data are presented as mean ± SD; *p < 0.05.

To examine the impact of DNA-PK in high glucose-induced DNA damage in CMECs *in vivo*, we employed a type 2 diabetes mouse model induced by STZ and HFD treatment. CMECs were isolated from the hearts. DNA-PK inhibitors NU7441 or M9831 were administered to inhibit DNA-PK complex activity. The STZ/HFD treatment led to a significant increase in the mRNA expression of crucial genes associated with DNA repair and cellular stress responses when compared to the NCD (Figure 6A-F). Notably, we observed a marked upregulation of Ku70 (Figure 6A), a pivotal component of the NHEJ pathway responsible for DBS break repair and a subunit of the DNA-PK complex. Similarly, the gene Brca1 (Figure 6B), essential for homologous recombination repair, was significantly increased. Additional genes involved in DNA repair, such as Xpc (Figure 6C), a factor in NER that removes bulky DNA adducts, and Ogg1 (Figure 6D), encoding the 8-oxoguanine DNA glycosylase for BER and oxidative DNA damage removal, were also upregulated.

In addition to DNA repair genes, The STZ/HFD treatment had a notable impact on the expression of genes involved in regulating the cell cycle and inducing apoptosis. We noted a significant increase in p21 mRNA levels (Figure 6E), which encodes a crucial cyclin-dependent kinase inhibitor, playing a key role in initiating cell cycle arrest in response to DNA damage. Furthermore, Bax (Figure 6F), a pro-apoptotic member of the Bcl-2 family, was upregulated, suggesting an enhanced apoptotic propensity in CMECs under diabetic conditions. Importantly, treatment with NU7441 or M9831, two structurally distinct DNA-PK inhibitors, significantly attenuated the STZ/HFD-induced gene upregulation, indicating that DNA-PK activity plays a central role in these transcriptional changes.

Consistent with these transcriptional alterations, STZ/HFD treatment also significantly increased Chk2 activity (Figure 6G), providing further evidence of DDR pathway activation. Additionally, we observed substantial elevation in γH2AX levels (Figure 6H) in CMECs from STZ/HFD-treated mice. γH2AX, the phosphorylated form of histone H2AX, is a widely used marker for DNA double-strand breaks, forming foci at damage sites. Finally, we measured 8-oxo-dG levels (Figure 6I), a biomarker of oxidative DNA damage resulting from ROS-induced guanine oxidation. 8-oxo-dG levels were markedly elevated in CMECs from STZ/HFD-treated mice, indicative of heightened oxidative stress. Crucially, treatment with NU7441 or M9831 significantly reduced Chk2 activity, γH2AX, and 8-oxo-dG levels, suggesting that DNA-PK inhibition mitigates high glucose-induced DNA damage. These results confirm that STZ/HFD-induced diabetes leads to enhanced DNA damage, including double-strand breaks and oxidative DNA lesions, and activates DDR pathways in cardiac microvascular endothelial cells.

### DNA-PK complex inhibition ameliorates cardiac dysfunction and endothelial barrier disruption in STZ/HFD-induced diabetic mice

**Figure 7:**
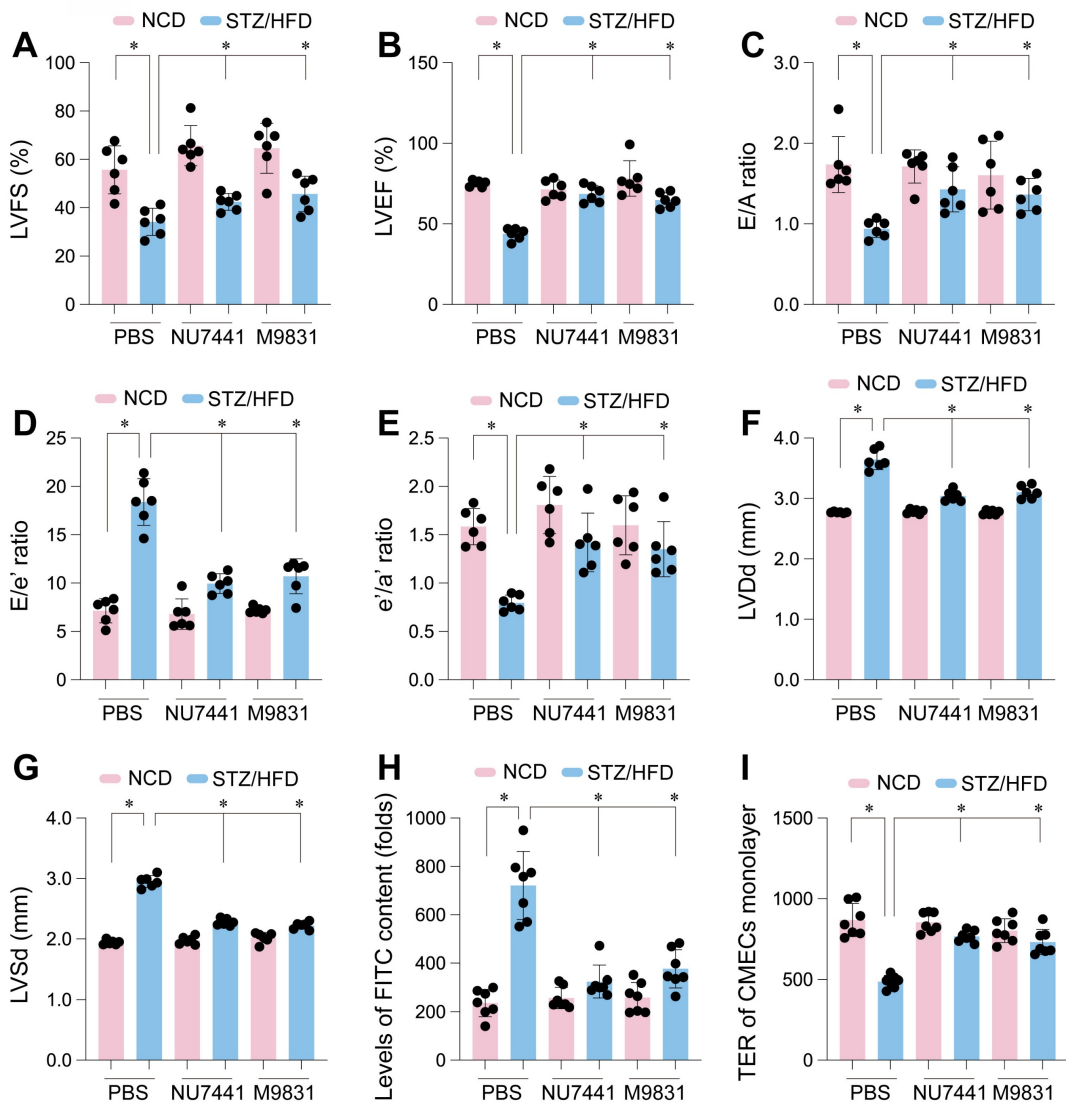
DNA-PK complex inhibition restores cardiac function and endothelial barrier integrity in diabetic mice. Cardiac microvascular endothelial cells (CMECs) were isolated from mice fed a normal control diet (NCD) or subjected to streptozotocin (STZ) and a high-fat diet (HFD) to induce type 2 diabetes. Mice received either PBS (vehicle), NU7441, or M9831 (DNA-PK inhibitors). (A-G) Assessment of cardiac function including (A) left ventricular fractional shortening (LVFS), (B) left ventricular ejection fraction (LVEF), (C) E/A ratio, (D) E/e' ratio, (E) e'/a' ratio, (F) left ventricular diastolic dimension (LVDd), and (G) left ventricular systolic dimension (LVSd). (H) FITC-dextran leakage assay in CMECs from diabetic hearts. (I) Transendothelial electrical resistance (TER) was measured in CMEC monolayers. Data are presented as mean ± SD; *p < 0.05.

To assess whether DNA-PK contributes to the cardiac dysfunction and endothelial barrier disruption in diabetic cardiomyopathy, we performed *in vivo* pharmacological inhibition. Mice with STZ/HFD-induced type 2 diabetes, exhibiting typical diabetic cardiomyopathy features, were treated with NU7441 or M9831, or vehicle control (PBS). Echocardiographic analysis showed that STZ/HFD treatment caused significant deterioration in left ventricular systolic function, reflected by substantial reductions in left ventricular fractional shortening (LVFS) and ejection fraction (LVEF) compared to NCD controls (Figure 7A-B). These parameters indicate impaired cardiac contraction, marking systolic dysfunction in the diabetic state. Additionally, STZ/HFD treatment induced diastolic dysfunction, a key feature of diabetic cardiomyopathy, as evidenced by decreased E/A ratio, reflecting impaired early diastolic filling, an increased E/e' ratio, signaling elevated left ventricular filling pressures, and a reduced e'/a' ratio (Figure 7C-E), indicative of impaired relaxation and increased ventricular stiffness. Furthermore, diabetes-induced structural remodeling was observed, as reflected by increased left ventricular dimensions at both end-diastole (LVDd) and end-systole (LVSd) (Figure 7F-G), suggesting diabetic-induced ventricular remodeling.

Remarkably, treatment with NU7441 or M9831 significantly improved these cardiac parameters. Both DNA-PK inhibitors restored LVFS and LVEF, reflecting the recovery of systolic function (Figure 7A-B). Likewise, the diastolic dysfunction indices (E/A, E/e', and e'/a' ratios) improved following DNA-PK inhibition, indicating reduced ventricular stiffness and enhanced relaxation (Figure 7C-E). The increased left ventricular dimensions were partially reversed by both inhibitors (Figure 7F-G), strongly suggesting that DNA-PK plays a crucial role in diabetic cardiomyopathy pathogenesis.

Given the established relationship between endothelial dysfunction and cardiac impairment in diabetes, we next evaluated the impact of DNA-PK inhibition on endothelial barrier integrity. We assessed endothelial permeability by measuring FITC-dextran leakage into endothelial cells isolated from diabetic hearts. STZ/HFD treatment led to significant FITC-dextran leakage, indicating compromised endothelial integrity (Figure 7H). To further quantify barrier function, we measured transendothelial electrical resistance (TER) in CMEC monolayers. STZ/HFD treatment significantly reduced TER, suggesting disrupted cell-cell junctions and compromised barrier function (Figure 7I). Notably, treatment with NU7441 or M9831 significantly reduced vascular permeability and restored TER towards control levels (Figure 7H-I), demonstrating that DNA-PK inhibition protects against endothelial barrier dysfunction. These findings support the vital influence of DNA-PK in the development of diabetic cardiomyopathy and associated microvascular complications, likely through mediating high glucose-induced DNA damage and cellular stress.

### Single-nucleus RNA sequencing identifies a high DNA damage response subpopulation of cardiac capillary endothelial cells in diabetic mice

**Figure 8:**
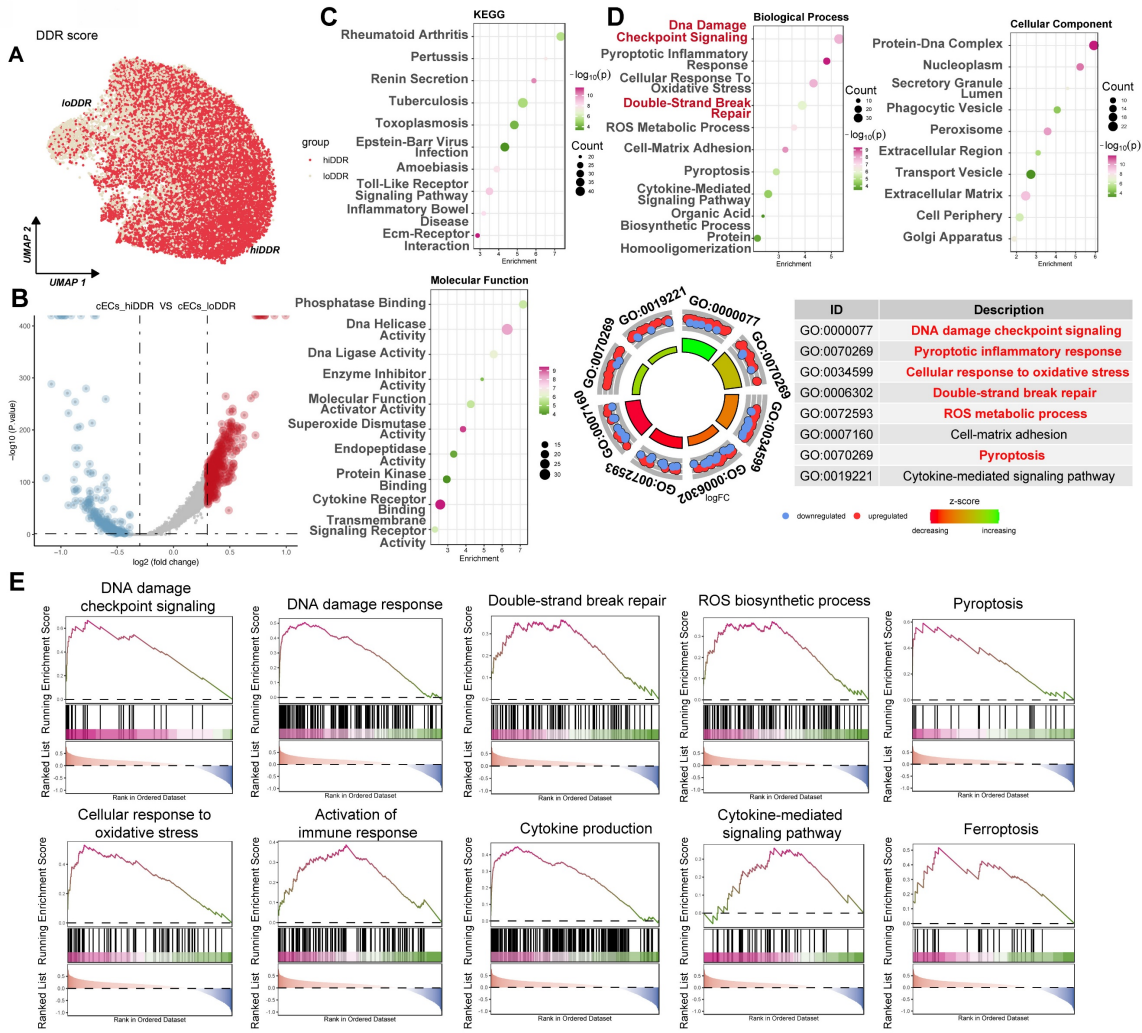
** Characterization of a high-DDR subpopulation of cardiac capillary endothelial cells in diabetic mice.** (A) UMAP plot. Capillary endothelial cells (cECs) from the CRA007245 dataset, colored by AUCell score for DDR. Cells are classified as 'hiDDR' (high DDR activity) or 'loDDR' (low DDR activity).(B) Volcano plot. Differential expression between hiDDR and loDDR cECs; red dots indicate significantly upregulated genes in the hiDDR group, blue dots denote downregulated genes. (C) Bubble plots (KEGG and MF GO). Enrichment of KEGG pathways (top) and Molecular Function GO terms (bottom) among genes upregulated in hiDDR cECs; bubble size reflects gene count and color represents -log₁₀(p-value). (D) Bubble and GO circle plots. Enrichment of Biological Process and Cellular Component GO terms (top) with a GO ring plot (bottom) summarizing log₂ fold change and z-score for selected terms. (E) GSEA plots. Display the enrichment of specific pathways in hiDDR versus loDDR cECs. The green curve shows the running enrichment score, with black bars marking gene positions in the ranked list; NES indicates the normalized enrichment score.

To further dissect the cellular heterogeneity of DDR in cardiac capillary endothelial cells (cECs), we analyzed the CRA007245 dataset and categorized cECs based on the median AUCell score for DDR activity. This stratification identified two distinct subpopulations: a high DDR activity ('hiDDR') group and a low DDR activity ('loDDR') group (Figure 8A). Differential gene expression analysis showed a set of genes significantly upregulated in the hiDDR cECs (Figure 8B, red points). Pathway enrichment analysis of these upregulated genes indicated significant enrichment in KEGG pathways such as 'Rheumatoid arthritis,' 'Toxoplasmosis,' and 'Toll-like receptor signaling' (Figure 8C, top). Molecular function GO terms enriched included 'Phosphatase Binding,' 'DNA Helicase Activity,' and 'DNA Ligase Activity' (Figure 8C, bottom). Further analysis revealed enrichment in biological processes such as 'DNA damage checkpoint signaling,' 'Cellular response to oxidative stress,' and 'Double-strand break repair,' as well as cellular components like 'Protein-DNA complex' and 'Nucleoplasm' (Figure 8D). A GO Circle plot summarized these findings, confirming the upregulation of pathways associated with DNA damage, oxidative stress, and inflammatory responses in the hiDDR cECs (Figure 8D). GSEA corroborated the enrichment of these pathways, with additional involvement of immune responses, cytokine production, and pyroptotic inflammation (Figure 8E). These results provide compelling evidence that a subpopulation of cardiac endothelial cells in diabetic mice exhibits a distinct transcriptional profile with heightened DDR activity and upregulation of genes associated with DNA repair, oxidative stress, and inflammation.

### DNA-PK complex inhibition attenuates ferroptosis and oxidative stress in CMECs of STZ/HFD-induced diabetic mice

**Figure 9:**
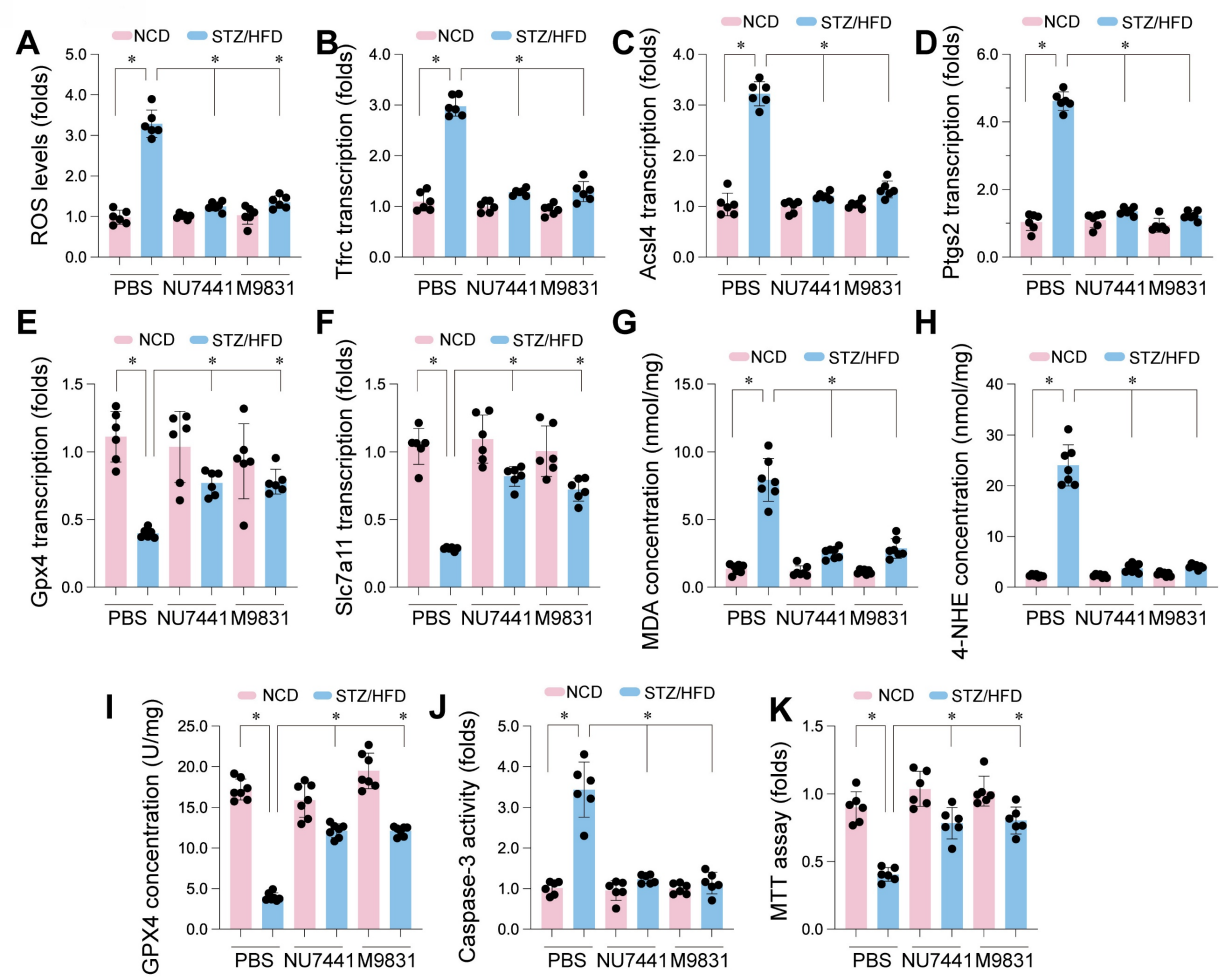
DNA-PK Complex inhibition mitigates ferroptosis and oxidative stress in CMECs from STZ/HFD-induced diabetic mice. Cardiac microvascular endothelial cells (CMECs) were isolated from mice fed a normal control diet (NCD) or subjected to streptozotocin (STZ) and a high-fat diet (HFD) to induce type 2 diabetes. Mice received either PBS (vehicle), NU7441, or M9831 (DNA-PK inhibitors). (A) Quantification of reactive oxygen species. (B-F) qPCR Analysis. Expression levels of genes regulating ferroptosis: (B) Tfrc, (C) Acsl4, (D) Ptgs2, (E) Gpx4, and (F) Slc7a11. (G) Measurement of malondialdehyde as a marker of lipid peroxidation. (H) 4-HNE concentration analysis. (I) GPX4 protein levels were assessed by western blotting. (J) Caspase-3 activity was measured by ELISA. (K) Cell viability was determined by the MTT assay. Data are presented as mean ± SD; *p < 0.05.

To explore whether DNA-PK contributes to ferroptosis in CMECs under diabetic conditions, the impact of DNA-PK inhibition on ferroptosis and oxidative stress markers was evaluated in mice with STZ/HFD-induced diabetes. STZ/HFD treatment significantly increased ROS levels compared to NCD controls, indicating elevated oxidative stress (Figure 9A). This ROS elevation was significantly reduced by NU7441 or M9831 treatment. We then analyzed genes regulating ferroptosis. STZ/HFD treatment significantly increased mRNA expression of Tfrc, Acsl4, and Ptgs2 (Figure 9B-D), pro-ferroptotic genes, and decreased mRNA expression of Gpx4 and Slc7a11 (Figure 9E-F), which protect against ferroptosis. Both NU7441 and M9831 reversed these changes, decreasing the expression of Tfrc, Acsl4, and Ptgs2, while increasing Gpx4 and Slc7a11 levels.

To further confirm ferroptosis involvement, we measured MDA and 4-HNE. STZ/HFD treatment elevated MDA and 4-HNE concentrations in CMECs (Figure 9G-H), indicating increased lipid peroxidation, a hallmark of ferroptosis. Consistent with gene expression data, STZ/HFD also significantly reduced GPX4 protein levels (Figure 9I). Importantly, treatment with NU7441 or M9831 significantly reduced MDA and 4-HNE levels and restored GPX4 protein expression, suggesting a protective role against lipid peroxidation and ferroptosis.

We also evaluated caspase-3 activity as a measure of apoptosis. STZ/HFD treatment significantly elevated caspase-3 activity (Figure 9J), which was markedly reduced by DNA-PK inhibition. Finally, the MTT assay revealed that STZ/HFD treatment reduced cell viability (Figure 9K), and this reduction was significantly rescued by treatment with DNA-PK inhibitors. These results demonstrate that STZ/HFD-induced diabetes leads to oxidative stress, increased ferroptosis susceptibility, lipid peroxidation, and reduced cell viability in CMECs. DNA-PK inhibition mitigates these effects, strongly implicating DNA-PK in regulating ferroptosis in CMECs under diabetic conditions.

## Discussion

Our study reveals a complex molecular cascade in type 2 diabetes mellitus (T2DM) cardiomyopathy, wherein hyperglycemia triggers a robust DNA damage response (DDR) that activates the DNA-PK complex—comprising PRKDC, XRCC5, and XRCC6—to drive endothelial ferroptosis and worsen microvascular dysfunction and cardiac decline. Through an integrated approach—combining transcriptomic analyses (GSE280770, GSE89475, GSE161931), single-nucleus RNA sequencing (CRA007245), and validation in the STZ/HFD mouse model—we observed substantial upregulation of DDR pathways, along with increased markers of DNA damage (γH2AX, 8-oxo-dG) and ferroptosis (elevated Tfrc, Acsl4, MDA, 4-HNE; reduced Gpx4, Slc7a11) in diabetic endothelial cells. Notably, pharmacological blockade of DNA-PK (NU7441 or M9831) ameliorated these detrimental effects by reducing oxidative stress, lipid peroxidation, and endothelial permeability, while restoring cardiac contractile (LVFS, LVEF) and relaxation (E/A, E/e') indices. These data implicate DNA-PK as a pivotal nexus linking genomic instability to iron-dependent cell death, underscoring a potential paradigm shift in therapeutic strategies for T2DM microvascular complications. By delineating cellular heterogeneity and mechanistic pathways, our findings not only enrich current knowledge but also hold considerable promise for clinical translation.

Mounting evidence supports a link between hyperglycemia-induced myocardial injury and DDR, though the precise downstream consequences remain debated. Rahmoon *et al.* 14 similarly demonstrated heightened DNA damage and DDR gene expression under high-glucose conditions, echoing our enrichment of DNA repair and DSB repair pathways. In parallel, another study 57 found that DDR activation—specifically ATM and γH2AX—is exacerbated by hyperglycemia in myocardial ischemia/reperfusion injury, consistent with our endothelial observations 56, 58, 59. However, Marsh *et al.* 60 implicate DDR in diabetic myocardial fibrosis, whereas Galati *et al.* 61 suggest a protective autophagic role, reflecting the longstanding uncertainty surrounding the pleiotropic effects of DDR. Our identification of DNA-PK-driven ferroptosis reframes DDR as a maladaptive cell-death mediator rather than a solely reparative or autophagic mechanism, thus offering a potential resolution to these conflicting findings 62, 63.

Although endothelial dysfunction under diabetic conditions is widely acknowledged, its mechanistic underpinnings remain incompletely understood 64-66. Recent investigations show that oxidative stress-induced DDR disrupts endothelial nitric oxide synthase (eNOS) function in diabetes 67 aligning with our observations of ROS-driven DDR. Another study 68 inks DDR to endothelial senescence in atherosclerosis, noting γH2AX accumulation comparable to our findings in diabetic CMECs. DDR has been posited as an adaptive mechanism that becomes maladaptive under chronic hyperglycemia, and our results—where DNA-PK inhibition rescues damage—support that view 14. In contrast, Pacher *et al.* 69 highlight a protective role for PARP1-mediated DDR in early diabetic endothelium, illustrating how the influence of DDR may vary over disease progression 70, 71. By pinpointing the DNA-PK-ferroptosis axis, our work advances beyond established models of senescence or eNOS impairment, offering a new dimension to DDR's pathological significance.

Despite clear evidence for hyperglycemia's harmful impact on myocardial endothelium, gaps remain regarding upstream regulators. Yang *et al.* 72 implicate ferroptosis in diabetic myocardial ischemia/reperfusion injury via endoplasmic reticulum stress, while Chen *et al.* 73 show that nicorandil mitigates microvascular ferroptosis through AMPK-Parkin-ACSL4—both consistent with our findings of elevated lipid peroxidation. A recent review 74 underscores iron overload in diabetic cardiomyopathy, resonating with our hiDDR subpopulation. Yet Wei *et al.* 75 describe endothelial dysfunction primarily through oxidative stress, overlooking DDR-ferroptosis interactions. Our data reveal DNA-PK as a master upstream conductor in this interplay, extending beyond the earlier work of Fok *et al.* 76—where DNA-PK inhibitors were deployed in cancer—and Ding *et al.* 77—which linked DNA-PK to hepatic ferroptosis in NAFLD 78. By centering on endothelial cells in diabetes, our results open new translational directions with heightened specificity.

These findings prompt several future lines of inquiry. Elucidating DNA-PK's interplay with ferroptosis mediators (e.g., GPX4, ACSL4) via proteomic screens or CRISPR-based perturbations could detail the fine structure of these signaling networks 79-81. Exploring DNA-PK's relevance across different microvascular beds—such as in diabetic nephropathy 82—would clarify its broader impact on diabetic complications. Larger animal models, akin to those employed by Fok *et al.* 83, could reinforce translational viability. Additionally, combining DNA-PK inhibition with established agents (e.g., SGLT2 inhibitors 84, antioxidants 85) may harness synergistic benefits. Finally, longitudinal human studies linking DNA-PK expression and ferroptosis markers could cement its role as both a therapeutic and prognostic target in diabetic microvascular disease.

Nonetheless, our approach carries certain limitations. While the STZ/HFD and db/db mouse models recapitulate many aspects of human T2DM, other common comorbidities, such as hypertension, may modulate DNA-PK dynamics 86, 87. Discrepancies in glucose concentrations (e.g., 20 mM in GSE280770 vs. 75 mM in GSE89475) could affect data interpretation, a common issue in diabetic research. Potential off-target effects of NU7441 and M9831—well-characterized in oncology but not fully assessed in this context—remain a consideration 88-90. In addition, single-nucleus RNA-seq might overlook cytoplasmic ferroptotic processes, reflecting an inherent constraint of snRNA-seq methods. Finally, short-term inhibitor administration precludes definitive statements on sustained benefit and safety, underscoring the need for longer-term studies resembling those in ferroptosis-targeted nephropathy research 91, 92.

Taken together, our work positions DNA-PK as a critical intermediary linking DDR and endothelial ferroptosis in T2DM cardiomyopathy, illuminating unresolved questions in both myocardial and endothelial pathophysiology. While our findings align with—and extend—existing DDR and ferroptosis literature, they also chart a path toward novel therapeutic avenues. Realizing these possibilities requires addressing the outlined constraints through further rigorous investigation.

## Supplementary Material

Supplementary table.

## Funding

This research was supported by Guangxi Natural Science Foundation (2023GXNSFAA026128). National Natural Science Foundation of China (NO.82360066; NO.82300315; NO.82374240), Guangdong Province Basic and Applied Basic Research Fund Project (No. 2024A1515012174; No.2024A1515013184). National Administration of Traditional Chinese Medicine Research Project (No. 0102023703), Project of the State Key Laboratory of Dampness Syndrome of Traditional Chinese Medicine jointly established by the province and the ministry (No.SZ2022KF10), Scientific Research Initiation Project of Guangdong Provincial Hospital of Traditional Chinese Medicine (No.2021KT1709), Research Project of Guangdong Provincial Bureau of Traditional Chinese Medicine (No.20241120), Guangdong Provincial Key Laboratory of Research on Emergency in TCM (No. 2023B1212060062; 2023KT15450), Excellent Young Talents Program of Guangdong Provincial Hospital of Traditional Chinese Medicine (No. SZ2024QN05) and Basic Clinical Collaborative Innovation Program of Guangdong Provincial Hospital of Traditional Chinese Medicine and School of Biomedical Sciences, The Chinese University of Hong Kong (No. YN2024HK01).
