# Supplementary material for: Hyperglycemia-induced DNA damage response activates DNA-PK complex to promote endothelial ferroptosis in type 2 diabetic cardiomyopathy
Source: Theranostics. 2025 Mar 19;15(10):4507–25. doi: 10.7150/thno.109514 (PMC11984385; doi:10.7150/thno.109514)
Supplement: Supplementary file 1 — Supplementary table. [file thnov15p4507s1.pdf]

Supplemental Tables

Supplemental Table 1. Primers for qPCR

Gene	Forward Primer	Reverse Primer
Mouse *Tfrc*	5'-TGGAGATCACAGAGGCAACC-3'	5'-GCTTCATCTTCAGGGTCAGG-3'
Mouse *Ftl*	5'-CAGCCTGTGGAGTTCTACGC-3'	5'-GGGTCACAGTGAGGTTGAGG-3'
Mouse *Acsl4*	5'-GGGAGACATCTATGCCACCA-3'	5'-TTCTCCACCTGCTTCTCCAC-3'
Mouse *Ptgs2*	5'-TGAGCAACTATTCCAAACCAGC-3'	5'-GCACGTAGTCTTCGATCACTATC-3'
Mouse *Gpx4*	5'-GCCTTCCCGTGTAACCAGTC-3'	5'-GGAGATAGCACGGCAGGTC-3'
Mouse *Slc7a11*	5'-ATGCTTCCTGGAGCCATTGG-3'	5'-CACAGCACTGCCAAGTCGTA-3'
